# Maternal and neonatal data collection systems in low- and middle-income countries for maternal vaccines active safety surveillance systems: A scoping review

**DOI:** 10.1186/s12884-021-03686-9

**Published:** 2021-03-17

**Authors:** Mabel Berrueta, Agustin Ciapponi, Ariel Bardach, Federico Rodriguez Cairoli, Fabricio J. Castellano, Xu Xiong, Andy Stergachis, Sabra Zaraa, Ajoke Sobanjo-ter Meulen, Pierre Buekens, Scoping Review Collaboration Group, Judith Absalon, Steve Anderson, Fernando Althabe, Shabir Madhi, Elizabeth McClure, Flor M. Munoz, Kissa W. Mwamwitwa, Annettee Nakimuli, Jennifer Clark Nelson, Lisa Noguchi, Lakshmi Panagiotakopoulos, Esperanca Sevene, Patrick Zuber, Maria Belizan, Eduardo Bergel, Alvaro Ciganda, Daniel Comande, Veronica Pingray

**Affiliations:** 1 Instituto de Efectividad Clínica y Sanitaria (IECS), Dr. Emilio Ravignani 2024 (C1014CPV), Buenos Aires, Argentina; 2 Tulane University School of Public Health and Tropical Medicine, New Orleans, LA 70112 USA; 3 University of Washington, Seattle, WA 98195-7631 USA; 4 Bill & Melinda Gates Foundation, Seattle, WA USA

**Keywords:** Active surveillance, Electronic Registries, Global Health, Health Information Systems, Pharmacovigilance, Pregnancy, Vaccination

## Abstract

**Background:**

Most post-licensure vaccine pharmacovigilance in low- and middle-income countries (LMICs) are passive reporting systems. These have limited utility for maternal immunization pharmacovigilance in LMIC settings and need to be supplemented with active surveillance. Our study’s main objective was to identify existing perinatal data collection systems in LMICs that collect individual information on maternal and neonatal health outcomes and could be developed to inform active safety surveillance of novel vaccines for use during pregnancy.

**Methods:**

A scoping review was performed following the Arksey and O’Malley six-stage approach. We included studies describing electronic or mixed paper-electronic data collection systems in LMICs, including research networks, electronic medical records, and custom software platforms for health information systems. Medline PubMed, EMBASE, Global Health, Cochrane Library, LILACS, Bibliography of Asian Studies (BAS), and CINAHL were searched through August 2019. We also searched grey literature including through Google and websites of existing relevant perinatal data collection systems, as well as contacted authors of key studies and experts in the field to validate the information and identify additional sources of relevant unpublished information.

**Results:**

A total of 11,817 records were identified. The full texts of 264 records describing 96 data collection systems were assessed for eligibility. Eight perinatal data collection systems met our inclusion criteria: Global Network’s Maternal Newborn Health Registry, International Network for the Demographic Evaluation of Populations and their Health; Perinatal Informatic System; Pregnancy Exposure Registry & Birth Defects Surveillance; SmartCare; Open Medical Record System; Open Smart Register Platform and District Health Information Software 2. These selected systems were qualitatively characterized according to seven different domains: governance; system design; system management; data management; data sources, outcomes and data quality.

**Conclusion:**

This review provides a list of active maternal and neonatal data collection systems in LMICs and their characteristics as well as their outreach, strengths, and limitations. Findings could potentially help further understand where to obtain population-based high-quality information on outcomes to inform the conduct of maternal immunization active vaccine safety surveillance activities and research in LMICs.

**Supplementary Information:**

The online version contains supplementary material available at 10.1186/s12884-021-03686-9.

## Background

Spontaneous or passive reporting systems are a cornerstone of vaccine safety surveillance in low- and middle-income countries (LMICs) [1]. This type of reporting relies on health professionals, patients, or others reporting suspected adverse events to public health or governmental organization. These systems have several limitations, including potentially inconsistent diagnostic criteria, underreporting, varying data quality, lack of data to establish a denominator, and little or no background information [1, 2]. In of these limitations, the launch of new vaccines for immunization of pregnant women requires additional safety surveillance efforts to be in place, including active surveillance in pregnant women and newborns and other post-approval safety monitoring mechanisms. Active surveillance aims to detect adverse events on an ongoing basis within a defined group of people. It is especially useful in conjunction with the introduction of new vaccines. Using active surveillance systems, new vaccines in development for maternal immunization (e.g., respiratory syncytial virus (RSV), group B streptococcus (GBS) and severe acute respiratory syndrome coronavirus 2 (SARS-CoV-2) could achieve their main goal of reducing morbidity and mortality in newborns in an informed manner [3]. Traditionally, active surveillance systems in LMIC have been linked to the Expanded Program on Immunization (EPI) and primarily focused on pediatric vaccines administered to children. For the evaluation of vaccine safety in pregnant women, maternal and neonatal data collection systems need to be leveraged to provide knowledge on background rates of pregnancy outcomes and newborn events. In the absence of an accurate background rate of an event, it is impossible to know if the adverse event is occurring at an expected or higher than expected rate. Having an established background rate would be further helpful for informing policies and designing active vaccine safety surveillance studies at sentinel sites [4, 5]. Perinatal outcomes information is generally unavailable in LMICs, due to many reasons, including the scarcity of resources and trained staff to support robust data collection systems, occurrence of vital and clinical events outside medical facilities, the lack of standardized, comprehensive, national registers and registration systems, inconsistencies among maternal newborn health outcome definitions, and the fact that medical records are often incomplete, poorly maintained and only paper based [1, 2, 6]. Lack of these records makes linking individual data from mother and their babies across systems cumbersome or sometimes impossible.

The Global Alignment of Immunization Safety Assessment in pregnancy (GAIA) project defined case definitions for main MNCH outcomes [7]. However, no sustainable answers are available on feasibility to implement them in the field [5, 7]. Various population-based surveys, surveillance systems, health information systems and perinatal data collection systems are already in place and could provide information on maternal and infant health in low-resource settings [8]. Mapping and harmonizing these existing platforms would allow LMICs to increase their ability to monitor relevant MNCH outcomes following maternal immunization.

There is an urgent need to develop or improve active safety surveillance of novel vaccines in pregnancy by understanding and adapting existing MNCH data collection systems. As part of a landscape analysis for integrated maternal immunization active safety surveillance and maternal data collection systems in LMICs, a scoping review was conducted to identify existing electronic and mixed paper-electronic data collection systems that register continuous and individual level MNCH data in LMICs with the potential to provide background data on diseases as well as record MNCH events/outcomes for active safety surveillance for novel maternal vaccines.

## Methods

We included studies describing electronic or mixed paper-electronic data collection systems in LMICs, including research networks, electronic medical records, and custom software platforms for health information systems. Medline PubMed, EMBASE, Global Health, Cochrane Library, LILACS, Bibliography of Asian Studies (BAS), and CINAHL were searched through August 2019. We also searched grey literature including through Google and websites of existing relevant perinatal data collection systems, as well as contacted authors of key studies and experts in the field to validate information and identify additional sources of relevant unpublished information.

We performed a scoping review following the Arksey and O’Malley six-stage approach [9] and the Preferred Reporting Items for Systematic Reviews and Meta-Analyses (PRISMA) statement guidelines and its extension for scoping reviews [10] (Additional file 1). The scoping review protocol was previously published in the Gates Open Research Journal [11].

Two main research questions guided the scoping review:

- *What existing prenatal and postnatal data collection systems are in place at the facility level and community level that could provide continuous, longitudinal, and individual information on maternal and neonatal health outcomes in LMICs?*
- *Do existing prenatal and postnatal data collection systems have the capacity to inform active safety surveillance for maternal vaccines and other maternal health interventions?*

Studies describing electronic or mixed paper-electronic perinatal data collection systems in LMICs, including research networks, electronic medical records, and custom software platforms for health information systems were included. Search strategies were run in databases (Medline, PubMed, EMBASE, Global Health, Cochrane Library, LILACS, Bibliography of Asian Studies (BAS), and CINAHL) and Google through August 2019. Grey literature including websites of existing data collection systems were explored [11].

**Fig. 1:**
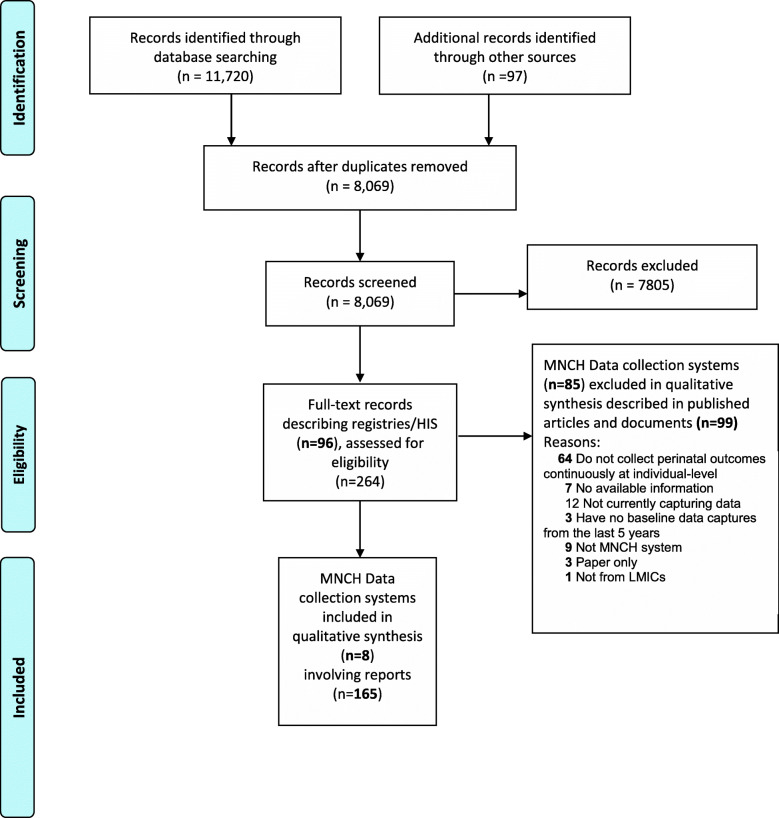
The PRISMA flow diagram details the selection process applied during the systematic literature search and review.

The PRISMA Extension for Scoping Reviews (PRISMA-ScR) flow diagram represents the formal literature review and screening process developed (Fig. 1). From those perinatal data collection systems identified in the full text article review, all specific data points published in the protocol [11] and listed in Table 1 were recorded*.*

**Table 1:** Data points abstracted from selected studies [11].

a) Countries where the systems operate	
b) The extent of data collection (e.g. community services, facilities services)	
c) The main purpose of the system d) Records linkage	
e) The level of implementation (e.g. local, national)	
f) Target population (e.g. specified subgroups or total population)	
g) The data collection form used (e.g. electronic, paper)	
h) Whether the data entered into the registry was primary data, or if the registry was based on secondary data from pre-existing sources.	
i) MNCHcare periods that the system registers	
j) Whether it collects the MNCH variables defined by the GAIA project	
k) Whether it collects socio-demographic data, obstetric information, pre-existing conditions of each women, and antenatal care outcome process.	
l) Type of code for classification of diseases used	
m) The individual data capture process (how and where it is done)	
n) Baseline data (pregnancy and outcomes) timeframe	
o) Capability to import, compile and export electronic data.	
p) Capability to link individual MNCH data with other registers(e.g. laboratory records, expense drug records, vaccine registers)	
q) Type of monitoring to assess data quality	
r) Whether it has a back-up policy.	
s) Availability of data collection tools and system documentation	
t) Maintenance and update process	
u) Whether it has data access policies,	
v) Type of privacy protection	
w) Funding or sponsor of each system	
x) Prior use in active surveillance or pharmacovigilance or post-marketing surveillance.	

A modified framework to assess and describe the final identified existing MNCH eligible health systems attributes was used based on frameworks available in the literature [12–14]. *Governance; System design; Data management; Data sources, Outcomes and Data* quality are the six domains used to present the extracted data points (Table 2).

**Table 2:** Framework: domains used to present the extracted data points

Domains	
**Governance**	Partnerships, both private and across government agencies, and coalitions; system policy and digital standards, including privacy and security; information and communication technology standards and documentation; geographical area of influence; primary purpose of the system (clinical care, research, information systems, mortality registries, insurance registries, birth registration)
**System design**	Infrastructure development and maintenance; multilanguage; capacity to compile and transfer electronic data; data access; records linkage and interoperability integration; type of platform to be used; what other systems may link to, and how the systems integrate or interoperate.
**Data management**	Data collection method used (paper, electronic, both, or not defined); Data entry: based on primary data entered into the registry or based on a secondary/duplicate data collection from existing source, standardized classification; who capture individual data (physician, patient)
**Data sources**	The scale of the implementation of the registry (national, district, local); health sector involved (private, public, social security), the specified population captured by the registry data collection (total population, only subgroups/select population); variables that collect; prior use in post marketing surveillance.
**Outcomes**	Maternal and perinatal outcomes based on GAIA project [15] Neonatal death; Congenital anomalies; Neonatal infections; Preterm birth; Stillbirth; Low birth weight; Small for gestational age; Respiratory distress; Failure to thrive; Microencephaly; Neonatal encephalopathy; Neonatal seizures; Neurodevelopmental delay. Maternal death; Fetal distress; Postpartum hemorrhage; Antenatal bleeding; Dysfunctional labour; Spontaneous abortion; Preeclamsia/Eclampsia; Foetal growth retardation; Gestational diabetes; Postpartum endometritis; Gestational Hypertension; Chorioamnionitis; Ectopic pregnancy; Premature preterm rupture of membranes; Preterm labor; Insufficient cervix
**Data quality**	Data quality assurance practices; data quality assessments.

The objectives of the consultation phase were to share preliminary findings and comparative analysis of final list of systems with experts in order to validate the domains used to describe the systems (Table 2) and the extracted data points (Table 1) from each system, as well as to identify additional grey sources of information.

## Results

### Study selection and characteristics

A total of 11,817 records including additional sources from reference lists and grey literature were identified. After removing duplicates, the 8,069 records left were screened by title and abstract, and 7,805 were considered irrelevant mainly because they were not related to MNCH data collection systems in LMICs. The full texts of 264 records describing 96 data collection systems were assessed for eligibility and finally, eight perinatal data collection systems (involving 165 reports) were included in qualitative synthesis (Figure 1). The included 165 reports were categorized as descriptive articles (51 published and 12 unpublished), published research studies related to the data collection systems (n=87), official system websites (n=7), user manuals or guides (n=3), and other web links that were not official systems websites (n=5). The most frequent reasons for excluding 99 reports were not collecting perinatal outcomes continuously at the individual-level (64), not currently capturing data (12) or not being a specific MNCH data collection system (9). The reasons for final exclusions of the potentially eligible systems are presented in Additional file 3*.*

The eight data collection systems finally selected were: 1) Global Network’s Maternal Newborn Health Registry (GN-MNHR), 2) International Network for the Demographic Evaluation of Populations and their Health (INDEPTH), 3) Perinatal Informatic System (SIP), 4) Pregnancy Exposure Registry & Birth Defects Surveillance (PER/BDS), 5) SmartCare, 6) Open Medical Record System (OpenMRS), 7) Open Smart Register Platform (OpenSRP) and 8) District Health Information Software 2 (DHIS 2) (see Table 3).

**Table 3:** Included data collection systems

	Data collection system	Description	Countries where serves to capture MNCH individual data	Number of pregnant women/deliveries registered
**Research network**	Global Network’s Maternal Newborn Health Registry	The Global Network’s Maternal Newborn Health Registry (MNHR) is *a prospective, population-based registry* of pregnancies at the Global Network sites. It started in 2008, enrolls and follows up on pregnant women and their newborns up to 42 days postpartum.	It captures data in six countries, including sites in Pakistan; Kenya; Zambia ; Democratic Republic of the Congo; Guatemala; and two in India.	370,000 mother and their infants have been enrolled so far
	International Network for the Demographic Evaluation of Populations and their Health	INDEPTH is a *network* of independent Health and Demographic Surveillance System (HDSS) *sites that carry out longitudinal research*. The INDEPTH Network Maternal, Newborn & Child Health Working Group (MNCH-WG) coordinate the surveillance of pregnancies and outcome tracking.	Bangladesh; Burkina Faso; Cote d’Ivoire; Ethiopia; Gambia; Ghana; Guinea-Bissau; India; Indonesia; Kenya; Malawi; Malaysia; Mozambique; Nigeria; Senegal; South Africa; Tanzania; Uganda; Vietnam	97,499 Estimated number of total births over five years (2012-2017) captured within the HDSS (only in 5 sites: Bandim; Matlab; Kintampo; Dabat; Igangamayuge)
**Electronical medical record**	Perinatal Informatic System	Perinatal Informatic System (“SIP”, by the acronym in Spanish: Sistema Informático Perinatal) by Pan American Health Organization (PAHO) is a *perinatal clinical record* that Ministries of health and maternity services (public and private) have adopted.	Cuba; El Salvador; Guatemala; Honduras; Nicaragua; Panama; Dominician Republic; Mexico; Argentina; Bolivia; Brazil; Chile; Colombia; Ecuador; Paraguay; Peru; Uruguay; Venezuela	Not Available
	Pregnancy Exposure Registry & Birth Defects Surveillance	PER/BDS is *an electronic medical record system* designed to collect data on pregnancy exposures during routine care.	South Africa	42,300 estimated deliveries per year in Gauteng and in Western Cape
	SmartCare	SmartCare is a portable, *integrated and electronical medical system* developed by Zambia's Ministry of Health	Zambia	Not Available
**Custom Software platform**	District Health Information Software 2 tracker	DHIS2 is a customize *platform* typically used as national health information system. Tracker module allows to collect pregnant and babies data and track longitudinally the progress of a patients over time.	Bangladesh; Liberia; West Bank and Gaza; South Africa	Not Available
	Open Medical Record System	OpenMRS is a *software platform* and a reference application which enables the design of a customized medical records system with no programming knowledge.	Uganda; Malawi; Rwanda; Lesotho; Kenya; Haiti	Not Available
	Open Smart Register Platform	It is *a mobile-first platform*, built to enable data-driven decision making at all levels of the health system with the following health modules: Reproductive, maternal, neonatal, and child health, childhood immunizations, tuberculosis, nutrition, malaria and early childhood development.	Indonesia; Pakistán	Not Available

Regarding the geographic distribution of the systems, although they were implemented in many different countries and districts, not all sites captured individual maternal and neonatal data. Therefore, we only included the sites that met the objectives and inclusion criteria of our study. DHIS 2 tracker, GN-MHNR and INDEPTH are in sub-Sahara Africa and South Asia. Additionally, GN-MNHR is also located in Latin America and the Caribbean (Guatemala), DHIS 2 is in the Middle East and North Africa (West Bank and Gaza), and INDEPTH is in East Asia and the Pacific (Indonesia, Malaysia and Vietnam). PER/BDS is located only in South Africa. SmartCare is in Zambia and OpenSRP is in Indonesia and Pakistan. Finally, OpenMRS is located in Uganda, Rwanda, Lesotho, Malawi, Kenya and Haiti.

Out of the 165 included reports: 86 (52.1%) were related to INDEPTH [16–101], 26 (15.7%) to Global Network [102–127], 24 (14.5%) to DHIS 2 [128–151], 9 (5.4%) to OpenMRS [152–160], 6 (3.6%) to SIP [161–166], 6 (3.6%) to OpenSRP [167–172], 4 (2.4%) to PER/BDS [173–176], and 4 (2.4%) to SmartCare [177–180].

### Major findings by identified domain

Following the analysis of the extracted data, the eight included systems’ synthetized results were presented in seven domains: *Governance; System design; System management; Data management; Data sources, Outcomes and Data quality* (Tables 4, 5, 6, 7, 8 and 9). Extracted data has been made available in a web-interactive App: http://safeinpregnancy.org/la_sc/table_by_domain.html#.

**Table 4:** Governance

Variable	1: DHIS2	2: GN MNHR	3: INDEPTH	4: OpenMRS	5: OpenSRP	6: SIP	7: PER/BDS	8: SmartCare
**Sponsor/funding**	Norad, PEPFAR, BMGF, CDC, GAVI, UNICEF, WHO, UiO	NICHD, BMGF	WHO, BMGF, NIH, Wellcome Trust, Harvard University, Sida, GSK, Children’s investment fund foundation, Comic relief, Hewlett Foundation	CDC, USAID, The Rockefeller Foundation	Wellcome Trust, UNICEF, WHO, UBS, Qualcomm, PATH	CLAP/PAHO	PEPFAR, WHO, South Africa National Department of Health	CDC
**Institution(s) in charge of system development and updates**	University of Oslo	RTI International	INDEPTH Network	Partners In Health, Regenstrief Institute, South African Medical Reserch Council	Interactive Health Solutions, WHO	CLAP/PAHO	National Department of Health South Africa	BroadReach Consulting LLC
**Data privacy protection**	Yes	Yes	Yes	Yes	Yes	No	Yes	Yes
**Description of the mechanism/Process**	Anonymized data	Anonymized data	Anonymized data	Anonymized data	Anonymized data	Not applicable	Anonymized data	Anonymized data
**External Protection (Cybersecurity)**	Yes	Yes	Yes	N/A	Yes	No	Yes	Yes
**Access Policies**	Yes	Yes	Yes	Yes	Yes	No	Yes	Yes
**Description of the mechanism/ process**	User/password	User/password	User/password	User/password	User/password	Not applicable	User/password	User/password
**Backup policies**	Yes	Yes	Yes	Yes	Yes	Yes	Yes	Yes
**Primary purpose of the system/data collection**	Clinical care	Research	Surveillance	Clinical care	Clinical care	Clinical care	Surveillance	Clinical care

**Table 5:** System design

Variable	1: DHIS2	2: GN MNHR	3: INDEPTH	4: OpenMRS	5: OpenSRP	6: SIP	7: PER/BDS	8: SmartCare
**Type of platform**	Web based	Local installation	Web based	Web based	Web based	Local installation	Web based	N/A
**Type of license**	Free/Open Source	Private	N/A	Free/Open Source	Free/Open Source	Free/Close Code	Free/Open Source	N/A
**Type of database**	Postgre SQL	SQL Server, Other	My SQL, SQL Server, Postgre SQL, Other	My SQL, SQL Server	My SQL, Other	Other	N/A	SQL Server
**Operating system in which it runs**	Windows, Android	Windows	Linux, Android	Windows, Linux	Android	Windows	N/A	Windows
**Data Encryption**	Yes	No	Yes	Yes	Yes	No	Yes	N/A
**Language**	English, Other	English, French, Spanish, Other	English, Other	English, Other	English, French, Other	English, French, Spanish, Other	English	English
**Data Capture Site**	Hospitals Clinics	Hospitals Clinics Community	Community	Hospitals Clinics Community	Hospitals Clinics Community	Hospitals Clinics	Hospitals Clinics	Hospitals Clinics
**Allow to export data**	Yes	Yes	Yes	Yes	Yes	Yes	Yes	Yes
**Records Linkage (Individual identifiers)**	Yes	Yes	Yes	Yes	Yes	Yes	Yes	Yes
**Ability to integrate with other data sources**	Yes/API	Yes	Yes	Yes/API	Yes	No	Yes	Yes
**Ability to integrate with National Health System databases**	Yes	No	Yes	Yes	Yes	No	Yes	Yes
**Ability to link with laboratory registries**	Yes	No	Yes	Yes	Yes	No	Yes	Yes
**Capacity to compile and transfer data**	Yes	Yes	Yes	Yes	Yes	Yes	Yes	Yes

N/A: Not available information

**Table 6:** Data management

Variable	1: DHIS2	2: GN MNHR	3: INDEPTH	4: OpenMRS	5: OpenSRP	6: SIP	7: PER/BDS	8: SmartCare
**Data Capture format**	Paper, Electronic	Paper, Electronic	Paper, Electronic	Paper, Electronic	Electronic	Paper, Electronic	Paper	Paper
**Standarized Classifications**	ICD10	No	ICD10	ICD10, SNOMED-ST, Other	N/A	ICD10	ICD10	N/A
**Who capture individual data**	Physician, Health provider, Patient	Physician, Health provider	Physician, Health provider	Health provider	Health provider, Patient	Physician, Health provider	Health provider	Physician, Health provider
**Has been used previously for any post marketing surveillance?**	N/A	No	Yes	N/A	N/A	N/A	No	N/A
**If yes, for what?**	Not applicable	Not applicable	Malaria treatments, Seasonal trivalent influenza vaccine, Heamophilus influenza type b vaccine, PREVENT proyect , other	Not applicable	Not applicable	Not applicable	Not applicable	Not applicable
**If yes what type of post marketing surveillance?**	Not applicable	Not applicable	Active surveillance	Not applicable	Not applicable	Not applicable	Not applicable	Not applicable

N/A: Not available information

**Table 7:** Data sources

Variable	1: DHIS2	2: GN MNHR	3: INDEPTH	4: OpenMRS	5: OpenSRP	6: SIP	7: PER/BDS	8: SmartCare
**The scale of the implementation of the registry**	Local, District, National, International	Local, District, National, International	Local, District, National, International	Local, District, National	District, National	Local, District, National, International	District	National
**Health sector involved**	Public, Private	Public, Private	Public, Private	Public	Public	Public	Public	Public
**Maternal sociodemographic variables included?**	Age, Education, Place of residence	Age, Education, Place of residence	Age, Education, Place of residence	Age, Place of residence	Age, Education, Place of residence	Age, Education, Place of residence	Age, Education, Place of residence	Age
**Data capture antenatal care visits**	Yes	Yes	Yes	Yes	Yes	Yes	Yes	Yes
**Timming for antenatal data collection**	At each visit	Retrospective	Retrospective	Retrospective, At each visit	At each visit	At each visit	At each visit	At each visit
**Antenatal care testing captured?**	Shypilis, HIV, Proteinuiria, Anemia	Shypilis, HIV, Anemia	Shypilis, HIV, Anemia	Shypilis, HIV, Proteinuiria, Anemia	Shypilis, HIV	Shypilis, HIV, Anemia	HIV	HIV
**Birth information**	Yes	Yes	Yes	Yes	Yes	Yes	Yes	Yes
**Previous drugs exposure**	For pre existing conditions, Pregnancy related	Pregnancy related	For pre existing conditions, Pregnancy related	Pregnancy related	Pregnancy related	Pregnancy related	For pre existing conditions, Pregnancy related	For pre existing conditions, Pregnancy related
**Drugs for preexisting diseases, which?**	Antiretroviral	Not applicable	Antimalarial	Not applicable	Not applicable	Not applicable	Antiepileptic, Antiretroviral, Analgesics, Antituberculosis, Neuropsychiatric, Antibiotics	Antiretroviral
**Pregnancy related drugs, which?**	Iron, Folic acid	Iron, Vitamins, Calcium, Antibiotics, Misoprostol	Iron, Folic acid, Vitamins	Iron, Folic acid	Iron, Folic acid	Iron, Folic acid, Corticoids, Magnesium sulphate	Iron, Folic acid, Vitamins	Iron, Vitamins, Calcium
**Vaccines exposure registries**	Pregnancy related, Other	Pregnancy related	Pregnancy related, Other	Pregnancy related	Pregnancy related, Other	Pregnancy related, Other	Pregnancy related	N/A
**Vaccines, which**	Influenza, Tetanus	Influenza, Tetanus	Influenza, Haemophilus influenzae type B, Other	Tetanus	Tetanus , Hepatitis B, BCG, Pentavalent	Influenza, Tetanus, Hepatitis B	Group B Streptococcus	N/A

N/A: Not available information

**Table 8:** Maternal outcomes

Variable	1: DHIS2	2: GN MNHR	3: INDEPTH	4: OpenMRS	5: OpenSRP	6: SIP	7: PER/BDS	8: SmartCare
**Maternal death**	Yes	Yes	Yes	Yes	Yes	Yes	Yes	Yes
**Preeclamsia/Eclampsia**	Yes	Yes	No	Yes	N/A	Yes	N/A	N/A
**Gestational hypertension**	N/A	Yes	Yes	N/A	N/A	N/A	N/A	N/A
**Foetal distress**	No	No	Yes	Yes	Yes	Yes	Yes	N/A
**Ectopic pregnancy**	No	Yes	No	N/A	N/A	N/A	N/A	N/A
**Postpartum hemorrhage**	Yes	Yes	Yes	Yes	Yes	Yes	Yes	N/A
**Spontaneous Abortion**	Yes	Yes	Yes	Yes	Yes	Yes	Yes	N/A
**Antenatal bleeding**	No	Yes	Yes	Yes	Yes	Yes	Yes	N/A
**Dysfunctional labour**	Yes	Yes	No	Yes	Yes	Yes	Yes	N/A
**Foetal growth retardation**	Yes	No	No	Yes	N/A	Yes	Yes	N/A
**Gestational diabetes**	Yes	No	No	No	Yes	Yes	N/A	N/A
**Post-partum endometritis**	N/A	Yes	No	No	Yes	Yes	N/A	N/A
**Chorioamnionitis**	N/A	No	No	No	Yes	Yes	N/A	N/A
**Premature preterm rupture of membranes**	No	No	No	N/A	N/A	N/A	N/A	N/A
**Preterm labor**	No	No	No	N/A	N/A	N/A	N/A	N/A
**Insufficient cervix**	No	No	No	N/A	N/A	N/A	N/A	N/A

N/A: Not available information

**Table 9:** Maternal outcomes

Variable	1: DHIS2	2: GN MNHR	3: INDEPTH	4: OpenMRS	5: OpenSRP	6: SIP	7: PER/BDS	8: SmartCare
**Neonatal death**	Yes	Yes	Yes	Yes	Yes	Yes	Yes	Yes
**Congenital anomalies**	Yes	Yes	Yes	No	Yes	Yes	Yes	N/A
**Neonatal infections**	Yes	Yes	Yes	No	Yes	Yes	Yes	N/A
**Preterm birth**	Yes	Yes	Yes	Yes	N/A	Yes	Yes	N/A
**Stillbirth**	Yes	Yes	Yes	Yes	N/A	Yes	Yes	N/A
**Low birth weight**	Yes	Yes	Yes	Yes	N/A	Yes	Yes	N/A
**Small for gestational age**	Yes	Yes	Yes	Yes	N/A	Yes	Yes	N/A
**Neonatal encephalopathy**	No	No	No	No	N/A	Yes	Yes	N/A
**Respiratory distress**	Yes	Yes	No	No	Yes	Yes	Yes	N/A
**Failure to thrive**	No	No	No	Yes	N/A	Yes	Yes	N/A
**Microencephaly**	No	No	No	No	Yes	Yes	Yes	N/A
**Neonatal seizures**	No	Yes	No	No	N/A	No	N/A	N/A
**Neurodevelopmental delay**	No	No	No	No	N/A	No	N/A	N/A

N/A: Not available information

#### Governance (Table 4)

These systems are supported by different categories of institutions [181] such as private foundations (e.g., Bill and Melinda Gates Foundation, Wellcome Trust, The Rockefeller Foundation, Children’s Investment Fund Foundation, Hewlett Foundation), governmental agencies in high-income countries (e.g., United States Agency for International Development (USAID), Centers for Disease Control and Prevention, Norad), global health initiatives (President’s Emergency Plan For AIDS Relief, The Global Alliance for Vaccines and Immunization), research councils (National Institutes of Health, National Institute of Child Health and Human Development, Medical Research Council), non-governmental organizations (Comic Relief), international organizations (World Health Organization /Pan America Health Organization, UNICEF), universities (Harvard University, University of Oslo), private sector organizations (GlaxoSmithKline, Qualcomm) and LMIC governments (South Africa National Department of Health) [14, 17, 37, 77, 103, 161, 178].

Some organizations were also responsible for the development and implementation of the included systems and are responsible for its optimal operability, such as the University of Oslo in the case of DHIS 2 [131], Partners in Health for OpenMRS [152], WHO for OpenSRP [170] and National Department of Health South Africa for PER/BDS [182] (Table 4). The majority of the systems demonstrated features that support the protection and privacy of collected information through anonymization of data, implementation of passwords before access, or external security (cybersecurity) [38, 77, 103, 131, 141, 154, 170, 177, 179]. Up to the search date, neither GN-MNHR nor SIP allowed for data encryption.

We were able to access the operating manuals, data forms, and documentation for six out of the eight systems [17, 103, 131, 152, 163, 170]; for SmartCare and PER/BDS these types of documents were not identified. Although most of the systems were designed for clinical care, some had been conceptualized for research such as GN-MNHR [106, 113, 119, 122, 123] or surveillance such as INDEPTH [46, 49, 77, 100] or PER/BDS [174]. Some of these were designed to satisfy more than one objective, and in the case of OpenMRS, this varied in different locations where the system is in place [130, 145, 152, 161, 170, 178].

#### System design (Table 5)

The type of license was free and open-source for four systems: DHIS 2 [131, 140], OpenSRP [170], OpenMRS [156] and PER/BDS [182]. SIP used a closed-code source [161] and GN-MNHR used a private license. Web-based platforms were the most frequently used [46, 49, 131, 140, 153, 156, 170]. However, the two systems GN-MNHR [103] and SIP [161, 163] still used local networks. No information on the type of license was recorded on SmartCare and INDEPTH.

Interoperability was assessed through the system’s ability to compile, transfer and export data, and integrate with other data sources, systems, individual and laboratory records, and/or national health record databases. The DHIS 2 [131, 132, 145, 146, 148], INDEPTH [17, 18, 21, 28, 35–38, 46, 58, 97, 100], SmartCare [177–180], OpenMRS [153, 154], OpenSRP [171] and PER/BDS [174] all have these capabilities. GN-MNHR [103, 108] and SIP [161, 163] systems showed lack of ability to link with National Health databases and clinical or laboratory records. All eight data collection systems demonstrated flexibility to add new variables.

Data were captured only at facilities in the system SIP [161], SmartCare [178] and PER/BDS [174, 182]. Data were captured both at the facility and community level for the systems DHIS 2 [144, 148], INDEPTH [49, 100], OpenMRS [152–154] and OpenSRP [170]. GD-MNHR only captured data at the community level [108, 113]. GD-MNHR only captured data at the community level [17, 33, 109, 123, 130, 131].

#### Data management (Table 6)

Although all of the included systems recorded data electronically [28, 49, 62, 85, 100, 108, 141, 145, 150, 152, 161, 169] and SmartCare [178, 179], used a mixed modality and initially captured data only on paper. Trained health providers, including nurses and doctors, collected the data in all systems. Only the DHIS 2 system through the MomConnect platform [140] allows pregnant women to enter information directly into the system through their smartphones. OpenSRP promotes a mobile health platform that allows health workers to register and track patient data [167].

GN-MNHR, INDEPTH, PER/BDS and SIP coordinated the data collection and validation across the sites [17, 103, 161, 182]. In contrast, DHIS 2, OpenMRS , OpenSRP and SmartCare offered a module and platform that each site can customize, modify, and adapt for use with total autonomy [131, 152, 170, 178].

The tenth revision of the International Classification of Diseases (ICD10) was used to code outcomes and conditions by more than half of the systems: DHIS 2 [130, 131], INDEPTH [100], OpenMRS [154], SIP [161] and PER/BDS [176]. No information was found regarding how OpenSRP and SmartCare systems classified and coded outcomes. The GN-MNHR system does not use any standardized classification. Only INDEPTH had been used for phase IV safety trials and post-marketing surveillance by a maternal health research platform [17, 183].

#### Data sources (Table 7)

All the systems can collect patient data and longitudinally track pregnant women’ progress and their babies over the prenatal and postnatal periods. However, timing of capturing information from antenatal visits is different between the eight systems. GN-MNHR and INDEPTH collected antenatal care data retrospectively [17, 103]. GN-MNHR collected their data at enrollment and delivery [103], and INDEPTH collected past events by self-reported data from household visits [17].

Drug exposures during pregnancy were recorded widely (e.g., antimalarial and antiretroviral treatment, iron, folic acid and vitamins) [17, 68, 103, 114, 127, 150, 152, 165, 170, 178, 182]. Exposure to vaccines was also collected, mainly of certain vaccines related to pregnancy (Influenza, tetanus/pentavalent) as well as non-pregnancy related vaccines (Hepatitis B, BCG, Haemophilus influenzae type B) [17, 68, 103, 114, 127, 130, 152, 165, 170, 178, 182]. PER/BDS system showed the widest drug and vaccine exposure recording, and intends to increase the list during the registry’ s future national implementation [174, 175]. We did not find information about collecting this information for SmartCare.

#### Maternal and neonatal outcomes (Tables 8 and 9)

Twenty-nine MNCH outcomes in selected data collection systems were searched: 16 maternal outcomes and 13 neonatal outcomes. We did not find information about SmartCare regarding their recorded perinatal outcomes.

All systems collected vital data such as maternal and neonatal deaths. The most frequently recorded perinatal outcomes were *fetal distress, postpartum hemorrhage, antenatal bleeding, dysfunctional labor, spontaneous abortion, congenital anomalies, neonatal infections, preterm birth, stillbirth, low birth weight, small for gestational age and respiratory distress*. Some outcomes were not recorded by any of the selected systems, i.e., premature preterm rupture of membranes, preterm labor, insufficient cervix and neurodevelopmental delay [184].

The seven systems with available data recorded 13 to 22 perinatal outcomes out of a total of 29 perinatal outcomes. Of the 16 maternal outcomes evaluated, SIP [161, 163, 165] and GN-MNHR [108, 110, 113, 119, 122] registered more than 50% of outcomes (n=11 and n=10 respectively) , DHIS 2 [131, 142, 150], OpenMRS [154] and OpenSRP [168–170] registered 50% of outcomes (n=8 each) and INDEPTH [38, 46, 49, 68, 77, 100] and PER/BDS [182] less than 50% of outcomes (n=6 each).

Of the 13 neonatal outcomes evaluated, SIP [161, 165], PER/BDS [175], GN-MNHR [105, 106, 108, 113, 114, 119], DHIS 2 [138, 145, 146, 150] and INDEPHT [38, 46, 49, 56, 68, 100] registered more than 50%, (n=11, n=11, n=10, n=9 and n=7 respectively) and OpenMRS [152, 154] and OpenSRP [169, 170] less than 50% (n=6 and n=5 outcomes, respectively).

#### Data quality (Table 10)

This domain was evaluated by examining information on both external and internal quality control mechanisms used by data collection systems. Internal monitoring was the most frequently cited procedure, specifically pre-programmed checks to avoid incorrect data entry [29, 38, 103, 139, 152, 156, 170]. Regarding external monitoring, only half of the systems reported having the necessary structures to be subject to frequent auditing and manual reporting [28, 38, 45, 108, 113, 145]. Only three systems demonstrated internal and external quality controls (DHIS 2, GN-MNHR and INDEPTH).

**Table 10:** Data Quality

Variable	1: DHIS2	2: GN MNHR	3: INDEPTH	4: OpenMRS	5: OpenSRP	6: SIP	7: PER/BDS	8: SmartCare
**External monitoring for data quality**	Yes	Yes	Yes	N/A	N/A	No	N/A	Yes
**If YES- Description of the mechanism/Process**	Weekly, monthly audits	Periodic audits, Monthly reposts	Periodic audits, Bi-weeky reports, Online data dashboard for real-time monitoring	Not applicable	Not applicable	Not applicable	Not applicable	Periodic source data verification
**Internal monitoring for data quality**	Yes	Yes	Yes	Yes	Yes	Yes	N/A	N/A
**If YES- Description of the mechanism/Process**	Pre-programmed data quality checks, Visual verification	Pre-programmed data quality checks	Pre-programmed data quality checks	Pre-programmed data quality checks	N/A	Pre-programmed data quality checks	Not applicable	Not applicable

N/A: Not available information

## Discussion

Through the present scoping review, 8,069 records were screened, and eight active data collection systems were identified. These systems continuously collect individual maternal and neonatal data in LMICs that can be leveraged for active safety surveillance of novel maternal vaccines.

Among the eight systems, seven systems are being used in countries in Africa, four in Asia and three in Latin America. Data collection systems served as research networks, perinatal electronic medical records, or a custom software platform for health information systems. The eight data collection systems showed variability regarding their governance, system design, data management, data sources, outcomes collected and data quality. Among these systems, all except SIP protected privacy of the information collected through anonymization of data. All systems except for GN-MNHR and SIP demonstrated interoperability capabilities and used web-based platforms. Data were recorded from antenatal care to postnatal period in all systems; however, GN-MNHR and INDEPTH collected antenatal visits data retrospectively. All systems collected vital data such as maternal and neonatal deaths as well as recorded exposure to vaccines and drugs during pregnancy. The most frequently recorded perinatal outcomes were *fetal distress, postpartum hemorrhage, antenatal bleeding, dysfunctional labor, spontaneous abortion, congenital anomalies, neonatal infections, preterm birth, stillbirth abortion, low birth weight, small for gestational age, respiratory distress and failure to thrive,* with variability among the systems. Any of the selected systems did not record the outcomes *premature preterm rupture of membranes, preterm labor, insufficient cervix, and neurodevelopmental delay*. GN-MNHR and INDEPTH coordinated the data collection and validation across their sites. In contrast, the rest of the systems offered a module and open-source platform that each site can customize, modify, and adapt for use with total autonomy. The tenth revision of the International Classification of Diseases (ICD10) was used for coding outcomes and conditions by DHIS 2, INDEPTH, OpenMRS, SIP and PER/BDS. No information was found on how outcomes and conditions were coded for the systems OpenSRP and SmartCare. As far as we know, GN-MNHR system does not use any standardized classification.

One close antecedent to our study is the work published by Froen et al., who, using WHO frameworks, mapped electronic registries (eRegistries) for maternal and child health [185]. The authors conducted a web-based survey of public health officials in LMICs and a search of literature from 2005 to 2015 to assess country capacity, quality and data usage in reproductive health registries. Froen et al. found 32 paper and electronic registry systems in 23 countries, supporting commonly used electronic and mobile applications for health. During those years, countries were in transition from paper-based data collection to electronic systems but very few have integrated electronic backbone systems. A more detailed framework was used to assess and describe the existing and eligible attributes of MNCH health systems, focusing on electronic data collection systems [12]. Our broad search conducted in August 2019 identified three times the number of registries (n=96). In contrast with their findings, we assessed that only eight were proficient in informing active safety vaccine surveillance system.

Zuber and colleagues [7] created a map of MNCH initiatives that collected health information to monitor maternal and child interventions in LMICs. The reported programs collected maternal and child health aggregate data and were fragmented in governance and financing and were duplicated in several related initiatives. They could not link individual-level data from pregnant women and their offspring including the linkage across individual records and multiple registers and sources. An active safety surveillance system for maternal vaccines would require statistics and monitoring of health data that reflect mother-baby dyad, characteristics, conditions and events from pregnancy to childbirth and postpartum care collected systematically, longitudinal, individual and uniform way. Our findings demonstrate that there are at least eight existing types of perinatal data collection systems/platforms implemented successfully in LMICS and can scale-up and collect MNCH individual-level data that track mothers and their babies.

Post-marketing surveillance of drugs used during pregnancy have been carried out in LMICs. Particularly, during the dolutegravir surveillance in Botswana (2018), 134 congenital abnormalities were identified in pregnant women exposed to this drug. Of these, the majority (104 cases) came from post-marketing studies, and only a few from spontaneous reports. However, although reporting post-marketing surveillance is useful, it lacks the ability to calculate prevalence rates because the true denominator is not usually available and births without defects are also underreported in LMICs frequently [186]. Another example, the International Maternal Pediatric Adolescents AIDS Clinical Trials (IMPAACT) network has been conducting clinical trials of drugs used during pregnancy with the aim of reducing perinatal transmission of human immunodeficiency virus. Some of them were phase IV trials and have provided important information during post-marketing stage. However, as limitation of those studies and as with most clinical trials data collection systems, some conclusions obtained might not be entirely extrapolated to the real world, and very low incidence adverse effects might not be detected in them [187]. Concerning antimalarial surveillance, a prospective observational study using HDSS (INDEPTH system) conducted in Burkina Faso, Kenya and Mozambique has evaluated artemisinin exposure and monitoring in pregnant women. Although the methods described and used in this study have been relevant in the development of pharmacovigilance of drugs in pregnancy and baseline perinatal prevalence rates might be measured in the regions, they have had certain limitations in quality and feasibility to collect certain outcomes. With the exception of the Kenya site where active surveillance has been carried out, in the other sites it was not possible to detect early miscarriages as well as the early identification of pregnancy [98].

The main strengths of our study are that we followed established methods [9, 188], utilized an exhaustive search strategy that included an in-depth grey literature search, and consulted large group of experts in the field with experience in pharmacovigilance, vaccine safety monitoring, as well as MNCH in LMICs on the results of this review.

The study’s main limitations are the heterogeneous and incomplete available reporting, forcing us to look for multiple non-peer-reviewed reports and directly contact authors and data system authorities to obtain a complete picture of each system. On-site visits and interviews to key referents could improve the completeness of this information, although these methods were beyond the scope of our protocol. For example, quality of data, capacity for data sharing and prevalence of maternal and infant health outcomes in each site from each system could also be obtained during future site visits in order to supplement the findings of this review.

Another limitation of our study is related to the definitions of maternal and neonatal outcomes that each system reported as being measured. The GAIA case definitions were used as a guide in order to ensure extracting all relevant perinatal data outcomes. Due to the fact that many of these GAIA definitions are really complex and require a lot of information to be considered as correctly defined, we cannot affirm that the full case definitions in each system comply exactly with the parameters proposed by GAIA [184].

Our findings have important implications not only for safety surveillance in maternal vaccines but also for policymakers and other stakeholders committed to research in MNCH. The analytical framework used demonstrated that all of the data collection systems identified in LMICs showed strengths and weaknesses to varying extents. However, several of the data collection systems are ready to inform future active safety surveillance. Regarding data protection, although most LMICs have not adopted a specific legislation or a Data Protection Authority [15], seven out of eight systems in this review included an appropriate data protection process to protect personal information about women and their children to be used, intentionally or otherwise, for purposes other than understanding and informing the prevention of poor health outcomes or to measure the safety of vaccines. Variability in case definitions and diagnostic criteria across data sources, and among differing cultures and languages was presented as a challenge by experts. Lack of harmonization of case confirmation/classification among systems was also a defined problem in LMICs [7, 189]. However, among the eight data collection systems found in our review, seven used the International Classification of Diseases (ICD) codes for standardized reporting of diseases. This is a promising finding to advance the integration and harmonization of the collection of MNCH data across systems in LMICs. Further in-depth exploration of these systems will provide more details about their capacity.

Our work could help to recognize and overcome the highlighted knowledge gap regarding the existence and capacity of surveillance platforms in LMICs for novel maternal vaccines. Identifying individual MNCH data platforms for pregnancy and disease surveillance is the first key action needed to identify potential sentinel sites for implementing integrated active surveillance successfully.

## Conclusion

We present a list of existing MNCH data capture systems in LMICs and describe in detail their characteristics, outreach, strengths, and limitations. This knowledge could potentially help policymakers, vaccine developers, researchers, and regulators to understand where to obtain population-based high-quality information on outcomes to inform and improve the conduct of vaccine active safety surveillance in LMICs.

## Supplementary Information

**Additional file 1:.** PRISMA Checklist. It contains the PRISMA Checklist.

**Additional file 2:.** Search strategy. It contains the search strategies used.

**Additional file 3:.** Data collection systems excluded. It contains the description of the excluded data collection systems.

## Data Availability

All data generated or analyzed during this study are included in this published article and its supplementary information files.

## Abbreviations

LMICS: Low- and middle-income countries
MNCH: Maternal neonatal and child health
GAIA: Global Alignment of Immunization Safety Assessment in pregnancy
WHO: World Health Organization
PRISMA: Preferred Reporting Items for Systematic Reviews and Meta-Analyses
GAVI: Global Alliance for Vaccines and Immunization
GN-MNHR: Global Network’s Maternal Newborn Health Registry
INDEPTH: International Network for Demographic Evaluation of Populations and their Health
SIP: Perinatal Informatic System
PER/BDS: Pregnancy Exposure Registry & Birth Defects Surveillance
OpenMRS: Open Medical Record System
OpenSRP: Open Smart Register Platform
DHIS 2: District Health Information Software 2
ICD10: The tenth revision of the International Classification of Diseases
