# Supplementary material for: Maternal and neonatal data collection systems in low- and middle-income countries for maternal vaccines active safety surveillance systems: A scoping review
Source: BMC Pregnancy Childbirth. 2021 Mar 17;21:217. doi: 10.1186/s12884-021-03686-9 (PMC7968860; doi:10.1186/s12884-021-03686-9)
Supplement: Supplementary file 2 — Additional file 2:. Search strategy. It contains the search strategies used. [file 12884_2021_3686_MOESM2_ESM.docx]

Additional file 2.

Search Strategy

**EMBase 23-08-2019**

| No. | Query | Results |
| --- | --- | --- |
| #44 | #43 AND (2012:py OR 2013:py OR 2014:py OR 2015:py OR 2016:py OR 2017:py OR 2018:py OR 2019:py) | 2936 |
| #43 | #15 AND #42AND #43 | 3866 |
| #42 | lmic:ti,ab OR subsaharan:ti,ab OR 'sub saharan':ti,ab OR 'southeast asia':ti,ab OR 'middle east':ti,ab OR 'central america':ti,ab OR 'south america':ti,ab OR caribbean:ti,ab OR afghanistan:ti,ab OR burundi:ti,ab OR benin:ti,ab OR burkina:ti,ab OR 'central african':ti,ab OR congo:ti,ab OR eritrea:ti,ab OR gambia:ti,ab OR 'guinea bissau':ti,ab OR madagascar:ti,ab OR mali:ti,ab OR 'sierra leone':ti,ab OR syria:ti,ab OR chad:ti,ab OR togo:ti,ab OR yemen:ti,ab OR bangladesh:ti,ab OR 'cote d ivoire':ti,ab OR cameroon:ti,ab OR comoros:ti,ab OR 'cabo verde':ti,ab OR egypt:ti,ab OR honduras:ti,ab OR indonesia:ti,ab OR india:ti,ab OR kyrgyz:ti,ab OR cambodia:ti,ab OR kiribati:ti,ab OR laos:ti,ab OR lesotho:ti,ab OR morocco:ti,ab OR myanmar:ti,ab OR mongolia:ti,ab OR nicaragua:ti,ab OR pakistan:ti,ab OR philippines:ti,ab OR papua:ti,ab OR gaza:ti,ab OR sudan:ti,ab OR senegal:ti,ab OR solomon:ti,ab OR salvador:ti,ab OR 'sao tome':ti,ab OR eswatini:ti,ab OR timor:ti,ab OR tunisia:ti,ab OR ukraine:ti,ab OR uzbekistan:ti,ab OR vietnam:ti,ab OR vanuatu:ti,ab OR zambia:ti,ab OR zimbabwe:ti,ab OR angola:ti,ab OR albania:ti,ab OR argentina:ti,ab OR armenia:ti,ab OR samoa:ti,ab OR azerbaijan:ti,ab OR bulgaria:ti,ab OR bosnia:ti,ab OR belarus:ti,ab OR belize:ti,ab OR bolivia:ti,ab OR brazil:ti,ab OR bhutan:ti,ab OR botswana:ti,ab OR china:ti,ab OR colombia:ti,ab OR 'costa rica':ti,ab OR cuba:ti,ab OR djibouti:ti,ab OR dominica*:ti,ab OR algeria:ti,ab OR ecuador:ti,ab OR ethiopia:ti,ab OR fiji:ti,ab OR micronesia:ti,ab OR gabon:ti,ab OR georgia:ti,ab OR ghana:ti,ab OR guinea:ti,ab OR grenada:ti,ab OR guatemala:ti,ab OR guyana:ti,ab OR haiti:ti,ab OR iran:ti,ab OR iraq:ti,ab OR jamaica:ti,ab OR jordan:ti,ab OR kazakhstan:ti,ab OR kenya:ti,ab OR lebanon:ti,ab OR liberia:ti,ab OR libya:ti,ab OR 'st. lucia':ti,ab OR 'sri lanka':ti,ab OR moldova:ti,ab OR maldives:ti,ab OR mexico:ti,ab OR 'marshal islands':ti,ab OR macedonia:ti,ab OR montenegro:ti,ab OR mozambique:ti,ab OR mauritania:ti,ab OR mauritius:ti,ab OR malawi:ti,ab OR malaysia:ti,ab OR namibia:ti,ab OR niger:ti,ab OR nigeria:ti,ab OR nepal:ti,ab OR nauru:ti,ab OR peru:ti,ab OR korea:ti,ab OR paraguay:ti,ab OR romania:ti,ab OR russia*:ti,ab OR rwanda:ti,ab OR somalia:ti,ab OR serbia:ti,ab OR suriname:ti,ab OR thailand:ti,ab OR tajikistan:ti,ab OR turkmenistan:ti,ab OR tonga:ti,ab OR turkey:ti,ab OR tuvalu:ti,ab OR tanzania:ti,ab OR uganda:ti,ab OR venezuela:ti,ab OR kosovo:ti,ab OR 'south africa':ti,ab | 1350239 |
| #41 | #16 OR #17 OR #18 OR #19 OR #20 OR #21 OR #22 OR #23 OR #24 OR #25 OR #26 OR #27 OR #28 OR #29 OR #30 OR #31 OR #32 OR #33 OR #34 OR #35 OR #36 OR #37 OR #38 OR #39 OR #40 | 2006876 |
| #40 | partum*:ti,ab | 17637 |
| #39 | parturition*:ti,ab | 15918 |
| #38 | childbirth*:ti,ab | 20093 |
| #37 | 'birth'/exp | 24807 |
| #36 | gestational:ti,ab | 149285 |
| #35 | perinatal:ti,ab | 90392 |
| #34 | 'perinatal care'/exp | 57564 |
| #33 | maternal:ti,ab | 304200 |
| #32 | mother*:ti,ab | 264544 |
| #31 | 'mother'/exp | 134002 |
| #30 | 'fetal':ti,ab | 296942 |
| #29 | 'fetus':ti,ab | 82530 |
| #28 | 'fetus'/exp | 198185 |
| #27 | 'newly born':ti,ab | 1419 |
| #26 | 'new born':ti,ab | 5144 |
| #25 | newborn*:ti,ab | 197195 |
| #24 | neonat*:ti,ab | 329389 |
| #23 | 'newborn'/exp | 576318 |
| #22 | pregnan*:ti,ab | 632176 |
| #21 | 'pregnancy'/exp | 741774 |
| #20 | 'ante natal':ti,ab | 783 |
| #19 | antenatal:ti,ab | 46163 |
| #18 | ('pre natal' NEAR/1 care*):ti,ab | 118 |
| #17 | (prenatal NEAR/1 care*):ti,ab | 11069 |
| #16 | 'prenatal care'/exp | 146094 |
| #15 | #1 OR #2 OR #3 OR #4 OR #5 OR #6 OR #7 OR #8 OR #9 OR #10 OR #11 OR #12 OR #14 | 347491 |
| #13 | pharmacovigilance:ti,ab AND system*:ti,ab AND vaccin*:ti,ab | 247 |
| #12 | 'outcome capture':ti,ab | 5 |
| #11 | 'data coordination':ti,ab | 75 |
| #10 | 'data hub':ti,ab | 37 |
| #9 | 'health information network':ti,ab | 261 |
| #8 | 'health information data':ti,ab | 72 |
| #7 | 'health statistics data':ti,ab | 101 |
| #6 | 'health information system':ti,ab | 2229 |
| #5 | 'medical information system'/exp | 19940 |
| #4 | 'e registr*':ti,ab | 97 |
| #3 | eregistr*:ti,ab | 15 |
| #2 | registr*:ti,ab | 297764 |
| #1 | 'register'/exp | 110298 |

**PubMed 23-08-2019**

|  | Query | Items found |
| --- | --- | --- |
| [#45](https://www.ncbi.nlm.nih.gov/pubmed/advanced) | (#16 AND #42 AND #43) Filters: Publication date from 2014/01/01 | [2938](https://www.ncbi.nlm.nih.gov/pubmed/?cmd=HistorySearch&querykey=44) |
| [#44](https://www.ncbi.nlm.nih.gov/pubmed/advanced) | (#16 AND #42 AND #43) | [6321](https://www.ncbi.nlm.nih.gov/pubmed/?cmd=HistorySearch&querykey=43) |
| #43 | (LMIC[tiab] OR "Low and Middle"[tiab] OR Subsaharan[tiab] OR Sub-Saharan[tiab] OR Southeast Asia*[tiab] OR Middle East*[tiab] OR Central America*[tiab] OR Caribbean[tiab] OR Afghanistan[tiab] OR Burundi[tiab] OR Benin[tiab] OR Burkina Faso[tiab] OR Central African[tiab] OR Congo[tiab] OR Eritrea[tiab] OR Ethiopia[tiab] OR Guinea[tiab] OR Gambia[tiab] OR Guinea-Bissau[tiab] OR Haiti[tiab] OR Liberia[tiab] OR Madagascar[tiab] OR Mali[tiab] OR Mozambique[tiab] OR Malawi[tiab] OR Niger[tiab] OR Nepal[tiab] OR Korea[tiab] OR Rwanda[tiab] OR Sierra Leone[tiab] OR Somalia[tiab] OR South Sudan[tiab] OR Syria[tiab] OR Chad[tiab] OR Togo[tiab] OR Tajikistan[tiab] OR Tanzania[tiab] OR Uganda[tiab] OR Yemen[tiab] OR Angola[tiab] OR Bangladesh[tiab] OR Bolivia[tiab] OR Bhutan[tiab] OR Cote d'Ivoire[tiab] OR Cameroon[tiab] OR Comoros[tiab] OR Cabo Verde[tiab] OR Djibouti[tiab] OR Egypt[tiab] OR Micronesia[tiab] OR Ghana[tiab] OR Honduras[tiab] OR Indonesia[tiab] OR India[tiab] OR Kenya[tiab] OR Kyrgyz[tiab] OR Cambodia[tiab] OR Kiribati[tiab] OR Laos[tiab] OR Lesotho[tiab] OR Morocco[tiab] OR Moldova[tiab] OR Myanmar[tiab] OR Mongolia[tiab] OR Mauritania[tiab] OR Nigeria[tiab] OR Nicaragua[tiab] OR Pakistan[tiab] OR Philippines[tiab] OR Papua[tiab] OR Gaza[tiab] OR Sudan[tiab] OR Senegal[tiab] OR Solomon[tiab] OR El Salvador[tiab] OR Sao Tome[tiab] OR Eswatini[tiab] OR Timor[tiab] OR Tunisia[tiab] OR Ukraine[tiab] OR Uzbekistan[tiab] OR Vietnam[tiab] OR Vanuatu[tiab] OR Zambia[tiab] OR Zimbabwe[tiab] OR Angola[tiab] OR Albania[tiab] OR Argentina[tiab] OR Armenia[tiab] OR Samoa[tiab] OR Azerbaijan[tiab] OR Bulgaria[tiab] OR Bosnia[tiab] OR Belarus[tiab] OR Belize[tiab] OR Bolivia[tiab] OR Brazil[tiab] OR Bhutan[tiab] OR Botswana[tiab] OR China[tiab] OR Cote d'Ivoire[tiab] OR Cameroon[tiab] OR Colombia[tiab] OR Comoros[tiab] OR Cabo Verde[tiab] OR Costa Rica[tiab] OR Cuba[tiab] OR Djibouti[tiab] OR Dominica[tiab] OR Dominican Republic[tiab] OR Algeria[tiab] OR Ecuador[tiab] OR Ethiopia[tiab] OR Fiji[tiab] OR Micronesia[tiab] OR Gabon[tiab] OR Georgia[tiab] OR Ghana[tiab] OR Guinea[tiab] OR Grenada[tiab] OR Guatemala[tiab] OR Guyana[tiab] OR Haiti[tiab] OR Iran[tiab] OR Iraq[tiab] OR Jamaica[tiab] OR Jordan[tiab] OR Kazakhstan[tiab] OR Kenya[tiab] OR Lebanon[tiab] OR Liberia[tiab] OR Libya[tiab] OR St. Lucia[tiab] OR Sri Lanka[tiab] OR Moldova[tiab] OR Maldives[tiab] OR Mexico[tiab] OR Marshall Islands[tiab] OR Macedonia[tiab] OR Montenegro[tiab] OR Mozambique[tiab] OR Mauritania[tiab] OR Mauritius[tiab] OR Malawi[tiab] OR Malaysia[tiab] OR Namibia[tiab] OR Niger[tiab] OR Nigeria[tiab] OR Nepal[tiab] OR Nauru[tiab] OR Peru[tiab] OR Korea[tiab] OR Paraguay[tiab] OR Romania[tiab] OR Russia*[tiab] OR Rwanda[tiab] OR Somalia[tiab] OR Serbia[tiab] OR Suriname[tiab] OR Thailand[tiab] OR Tajikistan[tiab] OR Turkmenistan[tiab] OR Tonga[tiab] OR Turkey[tiab] OR Tuvalu[tiab] OR Tanzania[tiab] OR Uganda[tiab] OR Venezuela[tiab] OR Kosovo[tiab] OR South Africa[tiab]) | [1058019](https://www.ncbi.nlm.nih.gov/pubmed/?cmd=HistorySearch&querykey=42) |
| [#42](https://www.ncbi.nlm.nih.gov/pubmed/advanced) | (#17 OR #18 OR #19 OR #20 OR #21 OR #22 OR #23 OR #24 OR #25 OR #26 OR #27 OR #28 OR #29 OR #30 OR #31 OR #32 OR #33 OR #34 OR #35 OR #36 OR #37 OR #38 OR #39 OR #40 OR #41) | [1794809](https://www.ncbi.nlm.nih.gov/pubmed/?cmd=HistorySearch&querykey=41) |
| [#41](https://www.ncbi.nlm.nih.gov/pubmed/advanced) | Partum[tiab] | [12448](https://www.ncbi.nlm.nih.gov/pubmed/?cmd=HistorySearch&querykey=40) |
| [#40](https://www.ncbi.nlm.nih.gov/pubmed/advanced) | Parturition*[tiab] | [14305](https://www.ncbi.nlm.nih.gov/pubmed/?cmd=HistorySearch&querykey=39) |
| [#39](https://www.ncbi.nlm.nih.gov/pubmed/advanced) | Childbirth*[tiab] | [16847](https://www.ncbi.nlm.nih.gov/pubmed/?cmd=HistorySearch&querykey=38) |
| [#38](https://www.ncbi.nlm.nih.gov/pubmed/advanced) | Parturition[Mesh] | [88780](https://www.ncbi.nlm.nih.gov/pubmed/?cmd=HistorySearch&querykey=37) |
| [#37](https://www.ncbi.nlm.nih.gov/pubmed/advanced) | Gestational[tiab] | [106116](https://www.ncbi.nlm.nih.gov/pubmed/?cmd=HistorySearch&querykey=36) |
| [#36](https://www.ncbi.nlm.nih.gov/pubmed/advanced) | Perinatal[tiab] | [68275](https://www.ncbi.nlm.nih.gov/pubmed/?cmd=HistorySearch&querykey=35) |
| [#35](https://www.ncbi.nlm.nih.gov/pubmed/advanced) | Perinatal Care[Mesh] | [9398](https://www.ncbi.nlm.nih.gov/pubmed/?cmd=HistorySearch&querykey=34) |
| [#34](https://www.ncbi.nlm.nih.gov/pubmed/advanced) | Maternal[tiab] | [242570](https://www.ncbi.nlm.nih.gov/pubmed/?cmd=HistorySearch&querykey=33) |
| [#33](https://www.ncbi.nlm.nih.gov/pubmed/advanced) | Mother*[tiab] | [207825](https://www.ncbi.nlm.nih.gov/pubmed/?cmd=HistorySearch&querykey=32) |
| [#32](https://www.ncbi.nlm.nih.gov/pubmed/advanced) | Mothers[Mesh] | [40363](https://www.ncbi.nlm.nih.gov/pubmed/?cmd=HistorySearch&querykey=31) |
| [#31](https://www.ncbi.nlm.nih.gov/pubmed/advanced) | Fetus[tiab] | [67461](https://www.ncbi.nlm.nih.gov/pubmed/?cmd=HistorySearch&querykey=30) |
| [#30](https://www.ncbi.nlm.nih.gov/pubmed/advanced) | Fetal[tiab] | [235782](https://www.ncbi.nlm.nih.gov/pubmed/?cmd=HistorySearch&querykey=29) |
| [#29](https://www.ncbi.nlm.nih.gov/pubmed/advanced) | Fetus[Mesh] | [154341](https://www.ncbi.nlm.nih.gov/pubmed/?cmd=HistorySearch&querykey=28) |
| [#28](https://www.ncbi.nlm.nih.gov/pubmed/advanced) | Newly Born*[tiab] | [1094](https://www.ncbi.nlm.nih.gov/pubmed/?cmd=HistorySearch&querykey=27) |
| [#27](https://www.ncbi.nlm.nih.gov/pubmed/advanced) | New Born*[tiab] | [4306](https://www.ncbi.nlm.nih.gov/pubmed/?cmd=HistorySearch&querykey=26) |
| [#26](https://www.ncbi.nlm.nih.gov/pubmed/advanced) | Newborn*[tiab] | [169507](https://www.ncbi.nlm.nih.gov/pubmed/?cmd=HistorySearch&querykey=25) |
| [#25](https://www.ncbi.nlm.nih.gov/pubmed/advanced) | Neonat*[tiab] | [255197](https://www.ncbi.nlm.nih.gov/pubmed/?cmd=HistorySearch&querykey=24) |
| [#24](https://www.ncbi.nlm.nih.gov/pubmed/advanced) | Infant, Newborn[Mesh] | [587396](https://www.ncbi.nlm.nih.gov/pubmed/?cmd=HistorySearch&querykey=23) |
| [#23](https://www.ncbi.nlm.nih.gov/pubmed/advanced) | Pregnan*[tiab] | [498458](https://www.ncbi.nlm.nih.gov/pubmed/?cmd=HistorySearch&querykey=22) |
| [#22](https://www.ncbi.nlm.nih.gov/pubmed/advanced) | Pregnancy[Mesh] | [865866](https://www.ncbi.nlm.nih.gov/pubmed/?cmd=HistorySearch&querykey=21) |
| #21 | Ante Natal[tiab] | [533](https://www.ncbi.nlm.nih.gov/pubmed/?cmd=HistorySearch&querykey=20) |
| [#20](https://www.ncbi.nlm.nih.gov/pubmed/advanced) | Antenatal[tiab] | [33352](https://www.ncbi.nlm.nih.gov/pubmed/?cmd=HistorySearch&querykey=19) |
| [#19](https://www.ncbi.nlm.nih.gov/pubmed/advanced) | Pre-Natal Care*[tiab] | [95](https://www.ncbi.nlm.nih.gov/pubmed/?cmd=HistorySearch&querykey=18) |
| [#18](https://www.ncbi.nlm.nih.gov/pubmed/advanced) | Prenatal Care*[tiab] | [10520](https://www.ncbi.nlm.nih.gov/pubmed/?cmd=HistorySearch&querykey=17) |
| [#17](https://www.ncbi.nlm.nih.gov/pubmed/advanced) | Prenatal Care[Mesh] | [25993](https://www.ncbi.nlm.nih.gov/pubmed/?cmd=HistorySearch&querykey=16) |
| #16 | (#1 OR #2 OR #3 OR #4 OR #5 OR #6 OR #7 OR #8 OR #9 OR #10 OR #11 OR #12 OR #13 OR #14 OR #15) | [325389](https://www.ncbi.nlm.nih.gov/pubmed/?cmd=HistorySearch&querykey=15) |
| #15 | (Pharmacovigilance[tiab] AND System*[tiab] AND Vaccin*[tiab] | [143](https://www.ncbi.nlm.nih.gov/pubmed/?cmd=HistorySearch&querykey=14) |
| [#14](https://www.ncbi.nlm.nih.gov/pubmed/advanced) | Outcome Capture*[tiab] | [9063](https://www.ncbi.nlm.nih.gov/pubmed/?cmd=HistorySearch&querykey=14) |
| [#13](https://www.ncbi.nlm.nih.gov/pubmed/advanced) | Data Coordination[tiab] | [39](https://www.ncbi.nlm.nih.gov/pubmed/?cmd=HistorySearch&querykey=13) |
| [#12](https://www.ncbi.nlm.nih.gov/pubmed/advanced) | Data Hub[tiab] | [25](https://www.ncbi.nlm.nih.gov/pubmed/?cmd=HistorySearch&querykey=12) |
| [#11](https://www.ncbi.nlm.nih.gov/pubmed/advanced) | “Health Information Network”[tiab] | [236](https://www.ncbi.nlm.nih.gov/pubmed/?cmd=HistorySearch&querykey=11) |
| [#10](https://www.ncbi.nlm.nih.gov/pubmed/advanced) | “Health Information Data”[tiab] | [44](https://www.ncbi.nlm.nih.gov/pubmed/?cmd=HistorySearch&querykey=10) |
| [#9](https://www.ncbi.nlm.nih.gov/pubmed/advanced) | “Health Statistics Data”[tiab] | [88](https://www.ncbi.nlm.nih.gov/pubmed/?cmd=HistorySearch&querykey=9) |
| [#8](https://www.ncbi.nlm.nih.gov/pubmed/advanced) | “Health Information System”[tiab] | [1715](https://www.ncbi.nlm.nih.gov/pubmed/?cmd=HistorySearch&querykey=8) |
| [#7](https://www.ncbi.nlm.nih.gov/pubmed/advanced) | Health Information Systems[Mesh] | [1135](https://www.ncbi.nlm.nih.gov/pubmed/?cmd=HistorySearch&querykey=7) |
| [#6](https://www.ncbi.nlm.nih.gov/pubmed/advanced) | Population Surveillance[Mesh] | [65437](https://www.ncbi.nlm.nih.gov/pubmed/?cmd=HistorySearch&querykey=6) |
| [#5](https://www.ncbi.nlm.nih.gov/pubmed/advanced) | Public Health Informatics[Mesh] | [1135](https://www.ncbi.nlm.nih.gov/pubmed/?cmd=HistorySearch&querykey=5) |
| [#4](https://www.ncbi.nlm.nih.gov/pubmed/advanced) | e-Registr*[tiab] | [12](https://www.ncbi.nlm.nih.gov/pubmed/?cmd=HistorySearch&querykey=4) |
| [#3](https://www.ncbi.nlm.nih.gov/pubmed/advanced) | eRegistr*[tiab] | [9](https://www.ncbi.nlm.nih.gov/pubmed/?cmd=HistorySearch&querykey=3) |
| [#2](https://www.ncbi.nlm.nih.gov/pubmed/advanced) | Registr*[tiab] | [214901](https://www.ncbi.nlm.nih.gov/pubmed/?cmd=HistorySearch&querykey=2) |
| [#1](https://www.ncbi.nlm.nih.gov/pubmed/advanced) | Registries[Mesh] | [87548](https://www.ncbi.nlm.nih.gov/pubmed/?cmd=HistorySearch&querykey=1) |

**Global Health (OVID) 23-08-2019**

| [# ▲](https://ovidsp-dc2-ovid-com.libproxy.tulane.edu/sp-3.33.0b/ovidweb.cgi?&S=DMIGFPDALFEBGMEEJPCKNGBFPKMKAA00&Sort+Sets=descending) | Searches | Results |
| --- | --- | --- |
| 1 | Registr*.ti,ab. | 26613 |
| 2 | eRegistr*.ti,ab. | 5 |
| 3 | e-Registr*.ti,ab. | 3 |
| 4 | Health Information System.ti,ab. | 593 |
| 5 | Health Statistics Data.ti,ab. | 25 |
| 6 | Health Information Data.ti,ab. | 18 |
| 7 | Health Information Network.ti,ab. | 24 |
| 8 | Data Hub.ti,ab. | 3 |
| 9 | Data Coordination.ti,ab. | 3 |
| 10 | Outcome Capture*.ti,ab. | 0 |
| 11 | (Pharmacovigilance and System* and Vaccin*).ti,ab. | 46 |
| 12 | or/1-11 | 27239 |
| 13 | exp Prenatal Care/ | 2618 |
| 14 | Prenatal Care*.ti,ab. | 3059 |
| 15 | Pre-Natal Care*.ti,ab. | 81 |
| 16 | Antenatal.ti,ab. | 12338 |
| 17 | Ante Natal.ti,ab. | 652 |
| 18 | exp Pregnancy/ | 96244 |
| 19 | Pregnan*.ti,ab. | 106392 |
| 20 | Neonat*.ti,ab. | 42876 |
| 21 | Newborn*.ti,ab. | 29720 |
| 22 | New Born*.ti,ab. | 1874 |
| 23 | Newly Born*.ti,ab. | 473 |
| 24 | exp Fetus/ | 16856 |
| 25 | Fetal.ti,ab. | 23189 |
| 26 | Fetus.ti,ab. | 8482 |
| 27 | exp Mothers/ | 33696 |
| 28 | Mother*.ti,ab. | 76267 |
| 29 | Maternal.ti,ab. | 65522 |
| 30 | Perinatal Care.ti,ab. | 403 |
| 31 | Gestational.ti,ab. | 21867 |
| 32 | Childbirth*.ti,ab. | 3991 |
| 33 | Parturition*.ti,ab. | 4033 |
| 34 | Partum.ti,ab. | 4300 |
| 35 | or/13-34 | 236731 |
| 36 | (lmic or subsaharan or 'sub saharan' or southeast asia or middle east or central america or south America or caribbean or afghanistan or burundi or benin or burkina or central african or congo or eritrea or ethiopia or guinea or gambia or 'guinea bissau' or haiti or liberia or madagascar or mali or mozambique or malawi or niger or nepal or korea or rwanda or sierra leone or somalia or sudan or syria or chad or togo or tajikistan or tanzania or uganda or yemen or angola or bangladesh or bolivia or bhutan or 'cote d ivoire' or cameroon or comoros or cabo verde or djibouti or egypt or micronesia or ghana or honduras or indonesia or india or kenya or kyrgyz or cambodia or kiribati or laos or lesotho or morocco or moldova or myanmar or mongolia or mauritania or nigeria or nicaragua or pakistan or philippines or papua or gaza or sudan or senegal or solomon or salvador or sao tome or eswatini or timor or tunisia or ukraine or uzbekistan or vietnam or vanuatu or zambia or zimbabwe or angola or albania or argentina or armenia or samoa or azerbaijan or bulgaria or bosnia or belarus or belize or bolivia or brazil or bhutan or botswana or china or colombia or costa rica or cuba or djibouti or dominica* or algeria or ecuador or ethiopia or fiji or micronesia or gabon or georgia or ghana or guinea or grenada or guatemala or guyana or haiti or iran or iraq or jamaica or jordan or kazakhstan or kenya or lebanon or liberia or libya or 'sri lanka' or moldova or maldives or mexico or marshal islands or macedonia or montenegro or mozambique or mauritania or mauritius or malawi or malaysia or namibia or niger or nigeria or nepal or nauru or peru or korea or paraguay or romania or russia* or rwanda or somalia or serbia or suriname or thailand or tajikistan or turkmenistan or tonga or turkey or tuvalu or tanzania or uganda or venezuela or kosovo or south Africa).ti,ab. | 678945 |
| 37 | 12 and 35 and 36 | 983 |
| 38 | limit 37 to yr="2014 -Current" | 476 |

**LILACS (BVS-iAH-EN) 30-07-2019**

| Search on : | (MH Registries OR Registro OR eRegistr$ OR MH Public Health Informatics OR MH Population Surveillance OR MH Health Information Systems) AND (MH Prenatal Care OR Prenatal OR Antenatal OR MH Pregnancy OR Pregnan$ OR Embaraz$ OR MH Infant, Newborn OR Neonat$ OR Newborn$ OR MH Fetus OR Fetal OR Fetus OR MH Mothers OR Mother$ OR Maternal OR MH Perinatal Care OR Perinatal OR Gestational OR Gestacional OR MH Parturition OR Childbirth$ OR Parturition$ OR Partum OR Parto) [Words] and 2014 OR 2015 OR 2016 OR 2017 OR 2018 OR 2019 [Country, year publication] | |
| --- | --- | --- |
| References found : | 474 [[refine](http://bases.bireme.br/cgi-bin/wxislind.exe/iah/online/#refine)] |  |

**Bibliography of Asian Studies (EBSCO) 23-08-2019**

| # | Query | Results |
| --- | --- | --- |
| S33 | S12 AND S31 Limiters - Year Published: 2014-2015 | 14 |
| S32 | S12 AND S31 | 7 |
| S31 | S13 OR S13 OR S14 OR S15 OR S16 OR S17 OR S18 OR S19 OR S20 OR S21 OR S22 OR S23 OR S24 OR S25 OR S26 OR S27 OR S28 OR S29 OR S30 | 4,348 |
| S30 | TX Partum | 34 |
| S29 | TX Parturition* | 10 |
| S28 | TX Childbirth* | 231 |
| S27 | TX Gestational | 8 |
| S26 | TX Perinatal | 20 |
| S25 | TX Maternal | 544 |
| S24 | TX Mother* | 2,688 |
| S23 | TX Fetus | 38 |
| S22 | TX Fetal | 40 |
| S21 | TX Newly Born* | 7 |
| S20 | TX New Born* | 497 |
| S19 | TX Newborn* | 49 |
| S18 | TX Neonat* | 62 |
| S17 | TX Pregnan* | 423 |
| S16 | TX Ante Natal | 1 |
| S15 | TX Antenatal | 33 |
| S14 | TX Pre-Natal Care* | 4 |
| S13 | TX Prenatal Care* | 27 |
| S12 | S1 OR S2 OR S3 OR S4 OR S5 OR S6 OR S7 OR S8 OR S9 OR S10 OR S11 | 662 |
| S11 | ( TI Pharmacovigilance AND TI System* AND TI Vaccin* ) OR ( AB Pharmacovigilance AND AB System* AND AB Vaccin*[tiab]) | 10 |
| S10 | TX Outcome Capture* | 2 |
| S9 | TX Data Coordination | 4 |
| S8 | TX Data Hub | 0 |
| S7 | TX Health Information Network | 16 |
| S6 | TX Health Information Data | 6 |
| S5 | TX Health Statistics Data | 7 |
| S4 | TX Health Information System* | 46 |
| S3 | TX e-Registr* | 2 |
| S2 | TX eRegistr* | 0 |
| S1 | TX Registr* | 589 |

**CINAHL (EBSCO) 23-08-2019**

| # | Query | Results |
| --- | --- | --- |
| S47 | S15 AND S43 AND S46 Limiters - Published Date: 20140101-20191231 | 2,330 |
| S47 | S15 AND S43 AND S46 | 3,348 |
| S46 | S44 OR S45 | 167,647 |
| S45 | AB (LMIC OR Subsaharan OR Sub-Saharan OR Southeast Asia OR Middle East OR Central America OR South America OR Caribbean OR Afghanistan OR Burundi OR Benin OR Burkina OR Central African OR Congo OR Eritrea OR Ethiopia OR Guinea OR Gambia OR Guinea-Bissau OR Haiti OR Liberia OR Madagascar OR Mali OR Mozambique OR Malawi OR Niger OR Nepal OR Korea OR Rwanda OR Sierra Leone OR Somalia OR Sudan OR Syria OR Chad OR Togo OR Tajikistan OR Tanzania OR Uganda OR Yemen OR Angola OR Bangladesh OR Bolivia OR Bhutan OR “Cote d Ivoire” OR Cameroon OR Comoros OR Cabo Verde OR Djibouti OR Egypt OR Micronesia OR Ghana OR Honduras OR Indonesia OR India OR Kenya OR Kyrgyz OR Cambodia OR Kiribati OR Laos OR Lesotho OR Morocco OR Moldova OR Myanmar OR Mongolia OR Mauritania OR Nigeria OR Nicaragua OR Pakistan OR Philippines OR Papua OR Gaza OR Sudan OR Senegal OR Solomon OR Salvador OR Sao Tome OR Eswatini OR Timor OR Tunisia OR Ukraine OR Uzbekistan OR Vietnam OR Vanuatu OR Zambia OR Zimbabwe OR Angola OR Albania OR Argentina OR Armenia OR Samoa OR Azerbaijan OR Bulgaria OR Bosnia OR Belarus OR Belize OR Bolivia OR Brazil OR Bhutan OR Botswana OR China OR Colombia OR Costa Rica OR Cuba OR Djibouti OR Dominica* OR Algeria OR Ecuador OR Ethiopia OR Fiji OR Micronesia OR Gabon OR Georgia OR Ghana OR Guinea OR Grenada OR Guatemala OR Guyana OR Haiti OR Iran OR Iraq OR Jamaica OR Jordan OR Kazakhstan OR Kenya OR Lebanon OR Liberia OR Libya OR St. Lucia OR Sri-Lanka OR Moldova OR Maldives OR Mexico OR Marshall Islands OR Macedonia OR Montenegro OR Mozambique OR Mauritania OR Mauritius OR Malawi OR Malaysia OR Namibia OR Niger OR Nigeria OR Nepal OR Nauru OR Peru OR Korea OR Paraguay OR Romania OR Russia* OR Rwanda OR Somalia OR Serbia OR Suriname OR Thailand OR Tajikistan OR Turkmenistan OR Tonga OR Turkey OR Tuvalu OR Tanzania OR Uganda OR Venezuela OR Kosovo OR South Africa) | 152,518 |
| S44 | TI (LMIC OR Subsaharan OR Sub-Saharan OR Southeast Asia OR Middle East OR Central America OR South America OR Caribbean OR Afghanistan OR Burundi OR Benin OR Burkina OR Central African OR Congo OR Eritrea OR Ethiopia OR Guinea OR Gambia OR Guinea-Bissau OR Haiti OR Liberia OR Madagascar OR Mali OR Mozambique OR Malawi OR Niger OR Nepal OR Korea OR Rwanda OR Sierra Leone OR Somalia OR Sudan OR Syria OR Chad OR Togo OR Tajikistan OR Tanzania OR Uganda OR Yemen OR Angola OR Bangladesh OR Bolivia OR Bhutan OR “Cote d Ivoire” OR Cameroon OR Comoros OR Cabo Verde OR Djibouti OR Egypt OR Micronesia OR Ghana OR Honduras OR Indonesia OR India OR Kenya OR Kyrgyz OR Cambodia OR Kiribati OR Laos OR Lesotho OR Morocco OR Moldova OR Myanmar OR Mongolia OR Mauritania OR Nigeria OR Nicaragua OR Pakistan OR Philippines OR Papua OR Gaza OR Sudan OR Senegal OR Solomon OR Salvador OR Sao Tome OR Eswatini OR Timor OR Tunisia OR Ukraine OR Uzbekistan OR Vietnam OR Vanuatu OR Zambia OR Zimbabwe OR Angola OR Albania OR Argentina OR Armenia OR Samoa OR Azerbaijan OR Bulgaria OR Bosnia OR Belarus OR Belize OR Bolivia OR Brazil OR Bhutan OR Botswana OR China OR Colombia OR Costa Rica OR Cuba OR Djibouti OR Dominica* OR Algeria OR Ecuador OR Ethiopia OR Fiji OR Micronesia OR Gabon OR Georgia OR Ghana OR Guinea OR Grenada OR Guatemala OR Guyana OR Haiti OR Iran OR Iraq OR Jamaica OR Jordan OR Kazakhstan OR Kenya OR Lebanon OR Liberia OR Libya OR St. Lucia OR Sri-Lanka OR Moldova OR Maldives OR Mexico OR Marshall Islands OR Macedonia OR Montenegro OR Mozambique OR Mauritania OR Mauritius OR Malawi OR Malaysia OR Namibia OR Niger OR Nigeria OR Nepal OR Nauru OR Peru OR Korea OR Paraguay OR Romania OR Russia* OR Rwanda OR Somalia OR Serbia OR Suriname OR Thailand OR Tajikistan OR Turkmenistan OR Tonga OR Turkey OR Tuvalu OR Tanzania OR Uganda OR Venezuela OR Kosovo OR South Africa) | 79,178 |
| S43 | S16 OR S17 OR S18 OR S19 OR S20 OR S21 OR S22 OR S23 OR S24 OR S25 OR S26 OR S27 OR S28 OR S29 OR S30 OR S31 OR S32 OR S33 OR S34 OR S35 OR S36 OR S37 OR S38 OR S39 OR S40 OR S41 OR S42 | 235,883 |
| S42 | TI Partum OR AB Partum | 2,081 |
| S41 | TI Parturition* OR AB Parturition* | 608 |
| S40 | TI Childbirth* OR AB Childbirth* | 9,706 |
| S39 | (MH "Labor+") | 11,869 |
| S38 | TI Gestational OR AB Gestational | 29,487 |
| S37 | TI Perinatal OR AB Perinatal | 18,421 |
| S36 | (MH "Perinatal Care") | 3,584 |
| S35 | TI Maternal OR AB Maternal | 62,919 |
| S34 | TI Mother* OR AB Mother* | 68,529 |
| S33 | (MH "Mothers+") | 35,340 |
| S32 | TI Fetus OR AB Fetus | 13,552 |
| S31 | TI Fetal OR AB Fetal | 34,919 |
| S30 | (MH "Fetus+") | 23,320 |
| S29 | TI Newly Born* OR AB Newly Born* | 366 |
| S28 | TI New Born* OR AB New Born* | 3,170 |
| S27 | TI Newborn* OR AB Newborn* | 25,443 |
| S26 | TI Neonat* OR AB Neonat* | 54,967 |
| S25 | TI Newborn* AND AB Newborn* | 4,776 |
| S24 | TI Neonat* AND AB Neonat* | 14,668 |
| S23 | (MH "Infant, Newborn+") | 121,083 |
| S22 | TI Pregnan* OR AB Pregnan* | 111,095 |
| S21 | (MH "Pregnancy+") | 183,840 |
| S20 | TI Ante Natal OR AB Ante Natal | 164 |
| S19 | TI Antenatal OR AB Antenatal | 11,584 |
| S18 | TI Pre-Natal Care* OR AB Pre-Natal Care* | 326 |
| S17 | TI Prenatal Care* OR AB Prenatal Care* | 6,923 |
| S16 | (MH "Prenatal Care") | 14,664 |
| S15 | S1 OR S2 OR S3 OR S4 OR S5 OR S6 OR S7 OR S8 OR S9 OR S10 OR S11 OR S12 OR S13 OR S14 | 150,971 |
| S14 | (TI Pharmacovigilance AND TI System* AND TI Vaccin* ) OR ( AB Pharmacovigilance AND AB System* AND AB Vaccin*[tiab]) | 1 |
| S13 | TI Outcome Capture* OR AB Outcome Capture* | 4,654 |
| S12 | TI Data Coordination OR AB Data Coordination | 3,596 |
| S11 | TI Data Hub OR AB Data Hub | 450 |
| S10 | TI Health Information Network OR AB Health Information Network | 3,811 |
| S9 | TI Health Information Data OR AB Health Information Data | 34,747 |
| S8 | TI Health Statistics Data OR AB Health Statistics Data | 9,237 |
| S7 | TI Health Information System* OR AB Health Information System* | 24,413 |
| S6 | (MH "Health Information Systems+") | 49,128 |
| S5 | (MH "Population Surveillance+") | 8,592 |
| S4 | TI e-Registr* OR AB e-Registr* | 71 |
| S3 | TI eRegistr* OR AB eRegistr* | 9 |
| S2 | TI Registr* OR AB Registr* | 74,424 |
| S1 | (MH "Registries, Disease") | 9,151 |

**Cochrane Library (Wiley) 23-08-2019**

| ID | Search | Hits | |
| --- | --- | --- | --- |
| #1 | MeSH descriptor: [Registries] explode all trees | | 902 |
| #2 | Registr*:ti,ab,kw | | 63100 |
| #3 | eRegistr*:ti,ab,kw | | 13 |
| #4 | e-Registr*:ti,ab,kw | | 13 |
| #2 | Registr*:ti,ab,kw | | 63100 |
| #3 | eRegistr*:ti,ab,kw | | 13 |
| #4 | e-Registr*:ti,ab,kw | | 13 |
| #5 | MeSH descriptor: [] explode all trees | | 0 |
| #6 | MeSH descriptor: [Population Surveillance] explode all trees | | 636 |
| #7 | MeSH descriptor: [Health Information Systems] explode all trees | | 11 |
| #8 | Health Information System*:ti,ab,kw | | 50816 |
| #9 | Health Statistics Data:ti,ab,kw | | 20057 |
| #10 | Health Information Data:ti,ab,kw | | 87873 |
| #11 | Health Information Network:ti,ab,kw | | 4154 |
| #12 | Data Hub:ti,ab,kw | | 371 |
| #13 | Data Coordination:ti,ab,kw | | 3811 |
| #14 | Outcome Capture*:ti,ab,kw | | 2173 |
| #15 | Pharmacovigilance;ti,ab,kw AND System*:ti,ab,kw AND Vaccin*:ti,ab,kw 0 | |  |
| #16 | #1 OR #2 OR #3 OR #4 OR #5 OR #6 OR #7 OR #8 OR #9 OR #10 OR #11 OR #12 OR #13 OR #15 | | 168395 |
| #17 | MeSH descriptor: [Prenatal Care] explode all trees | | 1352 |
| #18 | Prenatal Care*:ti,ab,kw | | 3713 |
| #19 | Pre-Natal Care*:ti,ab,kw | | 43 |
| #20 | Antenatal:ti,ab,kw | | 3844 |
| #21 | Ante Natal:ti,ab,kw | | 61 |
| #22 | MeSH descriptor: [Pregnancy] explode all trees | | 7354 |
| #23 | Pregnan*:ti,ab,kw | | 58587 |
| #24 | MeSH descriptor: [Infant, Newborn] explode all trees | | 15206 |
| #25 | Neonat*:ti,ab,kw | | 20533 |
| #26 | Newborn*:ti,ab,kw | | 25895 |
| #27 | New Born*:ti,ab,kw | | 1532 |
| #28 | Newly Born*:ti,ab,kw | | 132 |
| #29 | MeSH descriptor: [Fetus] explode all trees | | 1711 |
| #30 | Fetal:ti,ab,kw | | 10945 |
| #31 | Fetus:ti,ab,kw | | 5730 |
| #32 | MeSH descriptor: [Mothers] explode all trees | | 1602 |
| #33 | Mother*:ti,ab,kw | | 15992 |
| #34 | Maternal:ti,ab,kw | | 18923 |
| #35 | MeSH descriptor: [Perinatal Care] explode all trees | | 517 |
| #36 | Perinatal:ti,ab,kw | | 5304 |
| #37 | Gestational:ti,ab,kw | | 15742 |
| #38 | MeSH descriptor: [Parturition] explode all trees | | 393 |
| #39 | Childbirth*:ti,ab,kw | | 3635 |
| #40 | Parturition*:ti,ab,kw | | 363 |
| #41 | Partum:ti,ab,kw | | 1498 |
| #42 | #17 OR #18 OR #19 OR #20 OR #21 OR #22 OR #23 OR #24 OR #25 OR #26 OR #27 OR #28 OR #29 OR #30 OR #31 OR #32 OR #33 OR #34 OR #35 OR #36 OR #37 OR #38 OR #39 OR #40 OR #41 | | 95123 |
| #43 | (LMIC OR Subsaharan OR Sub-Saharan OR Southeast Asia OR Middle East OR Central America OR South America OR Caribbean OR Afghanistan OR Burundi OR Benin OR Burkina OR Central African OR Congo OR Eritrea OR Ethiopia OR Guinea OR Gambia OR Guinea-Bissau OR Haiti OR Liberia OR Madagascar OR Mali OR Mozambique OR Malawi OR Niger OR Nepal OR Korea OR Rwanda OR Sierra Leone OR Somalia OR Sudan OR Syria OR Chad OR Togo OR Tajikistan OR Tanzania OR Uganda OR Yemen OR Angola OR Bangladesh OR Bolivia OR Bhutan OR “Cote d Ivoire” OR Cameroon OR Comoros OR Cabo Verde OR Djibouti OR Egypt OR Micronesia OR Ghana OR Honduras OR Indonesia OR India OR Kenya OR Kyrgyz OR Cambodia OR Kiribati OR Laos OR Lesotho OR Morocco OR Moldova OR Myanmar OR Mongolia OR Mauritania OR Nigeria OR Nicaragua OR Pakistan OR Philippines OR Papua OR Gaza OR Sudan OR Senegal OR Solomon OR Salvador OR Sao Tome OR Eswatini OR Timor OR Tunisia OR Ukraine OR Uzbekistan OR Vietnam OR Vanuatu OR Zambia OR Zimbabwe OR Angola OR Albania OR Argentina OR Armenia OR Samoa OR Azerbaijan OR Bulgaria OR Bosnia OR Belarus OR Belize OR Bolivia OR Brazil OR Bhutan OR Botswana OR China OR Colombia OR Costa Rica OR Cuba OR Djibouti OR Dominica* OR Algeria OR Ecuador OR Ethiopia OR Fiji OR Micronesia OR Gabon OR Georgia OR Ghana OR Guinea OR Grenada OR Guatemala OR Guyana OR Haiti OR Iran OR Iraq OR Jamaica OR Jordan OR Kazakhstan OR Kenya OR Lebanon OR Liberia OR Libya OR St. Lucia OR Sri-Lanka OR Moldova OR Maldives OR Mexico OR Marshall Islands OR Macedonia OR Montenegro OR Mozambique OR Mauritania OR Mauritius OR Malawi OR Malaysia OR Namibia OR Niger OR Nigeria OR Nepal OR Nauru OR Peru OR Korea OR Paraguay OR Romania OR Russia* OR Rwanda OR Somalia OR Serbia OR Suriname OR Thailand OR Tajikistan OR Turkmenistan OR Tonga OR Turkey OR Tuvalu OR Tanzania OR Uganda OR Venezuela OR Kosovo OR South Africa):ti,ab,kw | | 72833 |
| #44 | #16 AND #42 AND #43 with Publication Year from 2014 to present, with Cochrane Library publication date from Jan 2014 to present, in Trials | | 2389 |

1. Gray Literature Strategy

The following strategy was applied in Google "maternal neonatal data collection systems LMICs". Due to the fact that it did not provide many records of interest, we proceeded to search for information within official websites of all data collection systems that had been identified through the main search strategy (both included and finally excluded). It was decided to enter the full name of the DCS identified, as a search strategy in Google. The same was done with the 96 identified systems.

We also look for information on key government, agency and non-governmental organization websites such as the World Health Organization (https://www.who.int), Maternal and Child Survival Program ([www.mcsprogram.org](http://www.mcsprogram.org)), Measure Evaluation (https://www.measureevaluation.org ), and also on website of LMIC National Ministries of Health.
